# The interaction between intestinal microenvironment and stroke

**DOI:** 10.1111/cns.14275

**Published:** 2023-06-12

**Authors:** Linna Zhao, Jie Xiao, Songlin Li, Yuying Guo, Rong Fu, Shengyu Hua, Yuzheng Du, Shixin Xu

**Affiliations:** ^1^ First Teaching Hospital of Tianjin University of Traditional Chinese Medicine Tianjin China; ^2^ National Clinical Research Center for Chinese Medicine Acupuncture and Moxibustion First Teaching Hospital of Tianjin University of Traditional Chinese Medicine Tianjin China; ^3^ Tianjin Key Laboratory of Translational Research of TCM Prescription and Syndrome Tianjin China; ^4^ Tianjin University of Traditional Chinese Medicine Tianjin China

**Keywords:** gut microbiota, intestinal immunity, intestinal microenvironment, neuroinflammation, stroke, treatment

## Abstract

**Background:**

Stroke is not only a major cause of disability but also the third leading cause of death, following heart disease and cancer. It has been established that stroke causes permanent disability in 80% of survivors. However, current treatment options for this patient population are limited. Inflammation and immune response are major features that are well‐recognized to occur after a stroke. The gastrointestinal tract hosts complex microbial communities, the largest pool of immune cells, and forms a bidirectional regulation brain‐gut axis with the brain. Recent experimental and clinical studies have highlighted the importance of the relationship between the intestinal microenvironment and stroke. Over the years, the influence of the intestine on stroke has emerged as an important and dynamic research direction in biology and medicine.

**Aims:**

In this review, we describe the structure and function of the intestinal microenvironment and highlight its cross‐talk relationship with stroke. In addition, we discuss potential strategies aiming to target the intestinal microenvironment during stroke treatment.

**Conclusion:**

The structure and function of the intestinal environment can influence neurological function and cerebral ischemic outcome. Improving the intestinal microenvironment by targeting the gut microbiota may be a new direction in treating stroke.

## Graphical abstract

This review describes the structure and function of the intestinal microenvironment and highlight its cross‐talk relationship with stroke. Improving the intestinal microenvironment may be a potential strategy for treating stroke.

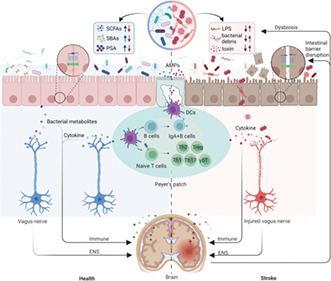

## 1 INTRODUCTION

Stroke is an acute cerebrovascular disease with cerebral ischemia and hemorrhagic injury as its main clinical features. Although stroke is the third leading cause of death after heart disease and cancer, it leads to permanent disabilities in 80% of survivors.^1^, ^2^ Ischemic stroke is usually caused by the occlusion of the great cerebral arteries and is the most common form of stroke, accounting for approximately 85% of all strokes.^3^, ^4^ After a stroke, patients can rapidly develop focal or global ischemic injury, which is highly disabling. There are currently two treatments for ischemic stroke: intravenous thrombolytic therapy with recombinant tissue prothrombin activator (tPA) and mechanical thrombectomy. However, both need to be performed within a limited time window. Therefore, new treatment strategies are urgently needed to improve the prognosis of stroke patients.

It has been established that after a stroke, up to 50% of patients develop gastrointestinal complications, including intestinal motility disorders, dysphagia, fecal incontinence, leaky gut, intestinal bleeding, and even enterogenic sepsis.^5^, ^6^ Stroke patients with gastrointestinal complications often have poor prognoses, increased mortality, and worsening neurological function.^7^, ^8^ The past decade has witnessed significant inroads achieved in the development of genomics, metabolomics, and proteomics, enabling research on the connection between the gut and stroke. The intestine is widely thought to be an important participant in the pathophysiological events after stroke and has become a research hotspot in recent years. Interestingly, almost 25% of T cells in the ischemic brain hemisphere are derived from the intestine.^5^ It is now understood that the brain and intestine form a complex “brain–gut axis” through various pathways,^9^, ^10^ which function in bidirectional regulation. Ischemic stroke alters the intestinal microenvironment leading to immune imbalance; conversely, the intestinal microenvironment can also influence stroke outcomes by modulating immune responses. However, research on the gut‐brain axis in stroke is still in its infancy, and understanding the intestinal microenvironment and its interaction with stroke could facilitate the development of new therapeutic strategies. In this review, we introduce the structure and function of the intestinal environment, provide a comprehensive overview of recent research advances in its interaction with stroke, and discuss possible therapeutic directions and principles.

## 2 INTESTINAL EPITHELIAL BARRIER AND STROKE

### 2.1 Normal structure and function of intestinal epithelial barrier

The intestinal epithelial barrier (IEB) is one of the largest interfaces between the outside world and the body's internal environment.^11^ The IEB is critical for maintaining intestinal homeostasis, serving as a physical barrier and a coordinating center of immune defense and bacterial‐immune cell dialogue.^12^ It has been shown that the IEB consists of four cell types: epithelial cells, goblet cells, Paneth cells, and enterochromaffin cells.^11^ Intestinal epithelial cells and goblet cells both produce mucin glycoproteins. Intestinal chromaffin cells are the most abundant neuroendocrine cells in the intestinal tract, and Paneth cells are responsible for the production of antimicrobial peptides,^13^, ^14^ which participate in resisting the invasion of pathogens. IEB secretes substances to form mucus that protects epithelial cells from bacteria, digestive enzymes, toxins, etc.^15^, ^16^ Current evidence suggests that mucus, mainly secreted by intestinal epithelial goblet cells, is highly glycosylated^17^ and plays an important role in maintaining the stability of the IEB. Mucus comprises 90–95% water, proteins, lipids, and electrolytes. Proteins are mainly produced by goblet cells, including mucins, which give mucus its gel‐like properties. Mucus also includes antimicrobial peptides and immunoglobulin A (IgA), allowing mucus to function as an innate defense.^18^ Interestingly, it has been shown that mucous fucosylation is significantly reduced in the presence of inflammation.^19^ The function of IEB depends on the presence of a series of intercellular junctions composed of apical junction complexes (AJC), including tight junctions (TJ) and adherent junctions (AJ), as well as desmosomes. The TJ structure is composed of transmembrane proteins located in the outer apical portion of the epithelial cell,^20^ preventing the passage of antigens through the IEB and playing a key role in maintaining barrier integrity. Adherent junctions and desmosomes are auxiliary structures for cell–cell adhesion located below TJ and are mainly composed of e‐cadherin, catenin, and actin filaments.^21^ Interestingly, adherent junctions establish cell–cell connections and promote the maturation of TJ. Moreover, TJ and AJ seal off epithelial cells and control the entry of gut microbes into the intestinal connective tissue.^22^ Desmosomes are composed of desmogleins and desmocollins, providing mechanical strength for cell‐to‐cell contact between epithelial cells.^23^

### 2.2 Structure and function changes to the intestinal epithelial barrier in stroke

**FIGURE 1:**
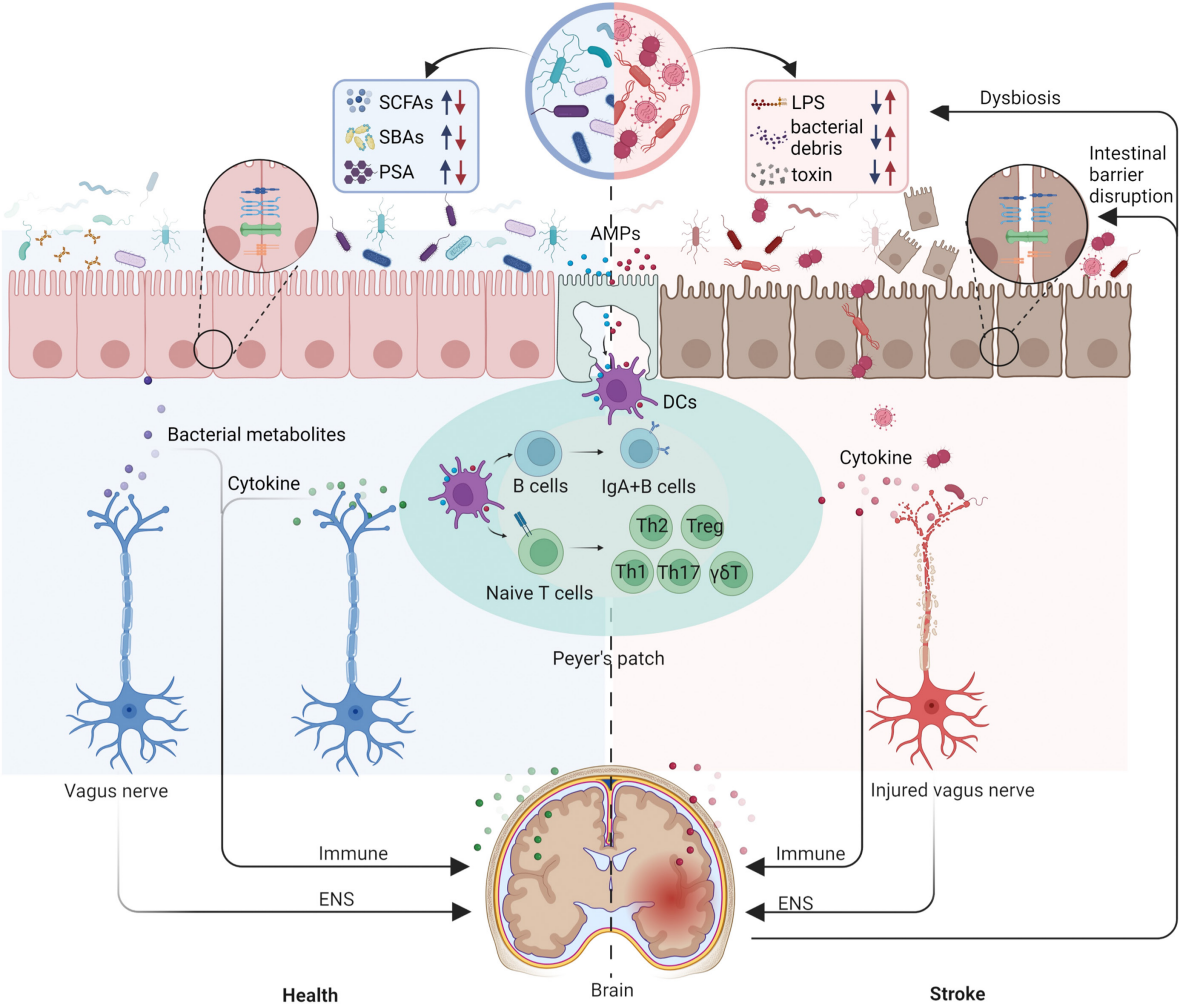
Routes of communication between the intestinal microenvironment and stroke. Reduced mucus layer and disrupted intercellular junctions in the IEB after stroke. The dysregulated gut microbiota produces low levels of SCFAs, Secondary bile acids (SBAs), and PSA, while producing high levels of LPS, bacterial debris, and toxin. Pathogenic bacteria have an increased opportunity to cross the damaged IEB, enter the intestinal lamina propria, and disrupt the balance of intestinal immunity. Subsequently, DCs acquire antigens and transport them to lymphoid tissue, where they initiate an adaptive immune response. The stress response to cerebral ischemic will affect the differentiation of T and B cells, which will secrete different cytokines. These cytokines further exacerbate intestinal inflammation and trigger apoptosis of enteric neurons. Some cytokines migrate with the peripheral circulation to the meninges, exacerbating ischemic neuroinflammation. Figure created with BioRender.com.

Current evidence suggests that stroke destroys the integrity of the IEB, resulting in intestinal villus epithelial injury, increased permeability, intestinal tight junction damage, reduced mucus, and enterogenic sepsis^14^, ^24^ (Figure 1). Liu et al. observed significant changes in small bowel morphology over time using a rat permanent middle cerebral artery occlusion (MCAO) model. At 6 h, epithelial cells on the tip of a few villi were necrotic and exfoliated. At 24 h, epithelial cell necrosis, exfoliation, and epithelial dissociation were observed in all villi.^25^ Dragana Stanley et al.^24^ found increased intestinal permeability in a mice model of MCAO 3 h after surgery, and the level of FITC detected in the blood was similar to that in a dextran sulfate sodium (DSS)‐induced mouse model of acute colitis. In another photochemically‐induced stroke mouse model, when the researchers assessed the intestinal barrier function, it was found that the intestinal permeability of mice increased 1 day after stroke, and ZO‐1, occludin, and claudin‐1 contents in the TJ were significantly decreased. Furthermore, TJ breakage was documented under an electron microscope.^26^ In addition, age affects the integrity of the IEB after stroke. Animal experiments have shown that stroke disrupts intestinal homeostasis more significantly in older mice than in younger ones.^27^, ^28^ Moreover, a study found that stroke in older mice leads to decreased goblet cells that secrete mucus, eventually leading to a deficiency in mucus production.^29^ Disruption of the integrity of the IEB after stroke can eventually result in microbiota translocation, whereby bacteria or bacterial components cross the barrier and enter the extra‐intestinal organs.^30^ Intriguingly, researchers administered a common commensal bacterium *Enterococcus faecalis* to germ‐free (GF) mice and examined its translocation and dissemination after experimental stroke induction. 24 h after colonization by *E. faecalis*, only GF mice that underwent MCAO showed bacterial translocation and spread to surrounding tissues such as the lung, liver, spleen, and mesenteric lymph nodes (MLNs).^24^

In conclusion, maintaining the integrity of the intestinal epithelial barrier (IEB) is crucial for preserving intestinal balance. Disruption of the IEB's structure and function following a stroke can result in microbial translocation, which may be a significant contributor to post‐stroke infections.

## 3 GUT MICROBIOME AND STROKE

### 3.1 Normal composition and function of gut microbiome

It is estimated that the human gut microbiota consists of 10^14^ bacteria, 10 times the number of cells in the human body. The gut commensal microbiome is a large and complex ecosystem characterized by mutual restraint and interdependence.^31^ The gut microbiota is mostly anaerobic, with a small fraction being aerobes and facultative anaerobes, archaea, viruses, and unicellular eukaryotes.^32^ So far, more than 50 phyla have been described, but the human gut microbiota is dominated by Bacteroidetes and Firmicutes, followed by Actinobacteria and Verrucomicrophyla.^32^, ^33^ The gut microbiota has diverse functions. In addition to promoting intestinal digestion, they also promote the integrity of the intestinal epithelial barrier by regulating TJ and AJ function,^34^, ^35^ the proliferation, differentiation, and migration of epithelial cells,^36^, ^37^ mucus secretion and antimicrobial peptide gene expression.^38^, ^39^ Current evidence suggests that the gut microbiota also assists in the development and function of the immune system. Peyer's patches composed of aggregated lymphoid follicles are dysplastic in the gut of GF mice. Compared with conventional mice and rats, the composition of CD4^+^ T cells and IgA‐producing B cells is altered in the lamina propria of the epithelial mucosal basal tissue in GF mice. Moreover, the gut microbiota in the intestinal lumen can enhance the induction of T lymphocyte subsets. For example, segmented filamentous bacteria (SFB) that adhere to the luminal surface of mouse intestinal Peyer's patches strongly stimulate Th17 differentiation in the lamina propria.^40^, ^41^ Gut commensal microbiota also regulates the development and function of lymphocytes in the small intestine, including Treg cells, γδT cells, Th17 cells, Th1 cells, and Th2 cells.^42^, ^43^, ^44^, ^45^, ^46^

In addition, the gut microbiota can affect the body through metabolites. These microbial metabolites are mainly divided into three categories, and the first type is produced by direct digestion or fermentation of food components by gut microbiota, such as short‐chain fatty acids (SCFAs). The fermentation of dietary fiber by anaerobic microorganisms in the small intestine produces SCFAs, which promote the secretion of intestinal mucus, regulate the permeability of the intestine,^47^, ^48^ provide energy for colonic muscle cells to treat high blood pressure,^49^ diabetes,^50^ multiple sclerosis (MS),^51^ emotional and cognitive dysfunction,^52^ and cardiovascular disease.^53^, ^54^ The second is metabolites produced by the host that enter the intestine to be reprocessed and modified by gut microbiota, such as secondary bile acids (SBAs). It is well‐established that gut microbiota can modify primary bile acids synthesized in the liver after entering the intestine with food. These modifications include the removal of amino acid residues through bile salt hydrolase (BSH) activity and further metabolism through dehydroxylation, dehydrogenation, or differential isomerization to produce SBAs.^55^ The microbiota involved in bile acid metabolism can alter the bioavailability and bioactivity of bile acids, thereby affecting epithelial cell proliferation, lipid and energy metabolism, and the expression of inflammatory genes.^56^, ^57^ The third is the self‐synthesized gut microbiota metabolites, such as polysaccharide A (PSA). PSA is a capsular polysaccharide mainly produced by nontoxigenic *Bacteroides fragilis* (NTBF) strains.^58^, ^59^ It induces CD4^+^ T cells to produce IL‐10 with an anti‐inflammatory effect by activating the TLR2/1 heterodimers and dectin‐1 signaling pathway on dendritic cells (DCs) and inhibits gastrointestinal inflammation.^60^, ^61^

### 3.2 Composition and function changes to gut microbiome in stroke

Overwhelming evidence substantiates that ischemic stroke alters the composition of the gut microbiota. In one study, 16S rDNA was used to sequence collected feces, and the results demonstrated increased levels of Bacteroidetes phylum following cerebral infarction in cynomolgus monkeys.^62^ It is widely acknowledged that *Prevotella*, belonging to the phylum Bacteroidetes, plays an essential pro‐inflammatory role in human chronic inflammatory diseases.^63^ Moreover, after cerebral infarction induction in cynomolgus monkeys, the increased relative abundance of the *Prevotella* genus may also be related to the post‐stroke inflammatory response.^62^ Reduced species diversity of gut microbiota and overgrowth of Bacteroidetes phylum 3 days after acute ischemic surgery in mice were identified as markers of post‐stroke microbiota dysbiosis.^5^ However, a case–control study showed reduced abundance levels of *Bacteroides*, *Prevotella*, and *Faecalibacterium* in patients with large‐artery atherosclerotic ischemic stroke and transient ischemic attack, and the microbial alpha diversity was increased.^64^ This result contradicts the findings observed in animal model experiments, which may be attributed to the fact that the daily diet of stroke patients and diseases such as hypertension, diabetes, and obesity may affect the gut microbiota.^65^, ^66^, ^67^, ^68^ In addition, *Faecalibacterium* and *Oscillospira* genera levels in monkey feces and butyrate concentrations decreased after 6–12 months of cerebral infarction induction.^62^ It is widely thought that *Faecalibacterium and Oscillospira* are the primary sources of butyrate production in the host.^69^, ^70^ Butyrate plays a critical role in maintaining the integrity of the intestinal barrier and inhibiting the production of pro‐inflammatory cytokines.^71^

Furthermore, gut microbiota could influence stroke outcomes through a bidirectional brain–gut communication pathway^5^, ^72^ (Figure 1). Benakis et al.^73^ performed MCAO surgery after pretreating mice with antibiotics and found that changes in gut microbiota reduced brain infarct size and improved sensorimotor function. Subsequently, Singh et al.^5^ revealed changes in the microbiota after cerebral ischemia and its role in neuroinflammatory response after stroke. To investigate this mechanism, they established a model of microbial transfer in GF mice. Microbiota obtained from sham‐operated and post‐filament middle cerebral artery occlusion model (fMCAO) mice were transplanted into GF recipient mice, and cortical damage was induced in GF recipient mice using the permanent distal MCA occlusion model (cMCAO). Recipients of the fMCAO group expressed high levels of pro‐inflammatory Th1 and Th17 cells in brain tissue to exacerbate stroke progression.^5^ Gut flora homeostasis was restored in recipients of the healthy group through fecal microbiota transplantation (FMT), increasing the abundance of Treg cells in the ischemic brain and significantly reducing brain damage.^5^

Microbial metabolites also play a crucial role in stroke recovery. Tryptophan (Trp) is an essential aromatic amino acid metabolized in the gut to indole, indole derivatives, and its ligands for the aryl hydrocarbon receptor (AHR).^74^ These tryptophan metabolites regulate the function of the intestinal barrier and immune cells through AHR signaling.^75^ In vivo studies in mice have shown that *Lactobacillus* amplification increases the production of indole‐3‐aldehyde (IAld), the molecule responsible for the ligand activity of the tryptophan metabolite AHR, which then maintains intestinal mucosal immune homeostasis via the AhR‐IL‐22 axis.^76^ Tryptophan concentrations have also been found to correlate with neuroinflammation and prognostic outcomes in acute ischemic stroke in clinical samples.^77^ Besides, short‐chain fatty acids can affect stroke recovery. To assess the potential correlation of gut microbiota and fecal SCFAs profiles with stroke prognosis in acute ischemic stroke (AIS) patients, researchers designed a prospective observational study of 140 AIS patients and 92 healthy individuals. Fecal bacterial counts and SCFA levels were determined by 16S rRNA gene sequencing and gas chromatography–mass spectrometry.^78^ These results confirm that patients with AIS (especially those with increased stroke severity) have a significant gut microbiota imbalance with SCFAs deficiency, which may trigger a leaky gut.^78^ A study conducted in Japan substantiated that patients with ischemic stroke had decreased acetic acid and increased valeric acid levels.^79^ Transplantation of SCFAs‐enriched fecal microbiota or the use of butyrate to influence SCFAs levels to repair leaky gut is reportedly effective for treating ischemic stroke.^80^ Notably, SCFA supplementation before stroke in mice improved behavioral function and cortical network plasticity at later stages after stroke. This effect may be due to microglia‐mediated activation by SCFAs via circulating lymphocytes.^81^ In another study, oral administration of SCFAs‐producing bacteria and inulin (the bacterial substrate produced by SCFAs) could reduce the percentage of IL‐17^+^γδT cells in ischemic brains and neurological deficits after stroke in older mice and improve depression‐like behavior compared with young mice.^82^ In addition, the blood–brain barrier (BBB) plays a crucial role as the brain's gatekeeper in maintaining the homeostasis of the central nervous system and normal neuronal function. One of the pathological features of stroke is BBB dysfunction, associated with disrupted TJ structure and increased BBB permeability, thereby increasing the risk of vasogenic edema and hemorrhagic transformation in patients, leading to poor outcomes.^83^, ^84^ Recent studies have found that butyrate improves BBB dysfunction and reduces BBB permeability after ischemic stroke in aged mice. This therapeutic effect may be attributed to the increased expression of IL‐22 in the brains of post‐stroke mice after butyrate treatment.^85^

In summary, the gut microbiota and its metabolites play a critical role in maintaining the IEB's integrity, as well as in the development and function of intestinal immune cells. Dysbiosis of the gut microbiota due to stroke can increase the number of pathogenic bacteria and alter several essential bioactive metabolites. Some of these metabolites can aid in neurological recovery, improve stroke prognosis, and may represent a novel approach to stroke injury repair.

## 4 INTESTINAL IMMUNE SYSTEM AND STROKE

### 4.1 Normal structure and function of the intestinal immune system

Gut‐associated lymphoid tissue (GALT) is considered the largest immune organ in the human body. There are three main types of GALT, Peyer's patches (PPs), MLNs, and isolated lymphoid follicles (ILFs).^86^ PPs consist of B cell follicles surrounding the T cell zone and surrounded by specialized epithelial cells.^87^ T cells in the PPs can migrate to MLNs and present antigens there.^88^ These PPs and MLNs activate lymphocytes (T cells and B cells) that can enter the peripheral circulation through the thoracic duct, generate an immune response, limit systemic inflammation, and lymphatically nest back into the lamina propria of the gut.^89^, ^90^ ILFs include other smaller cell populations, such as DCs, lymphoid tissue‐inducing cells (LTi), and large populations of B cells.^86^ DCs are antigen‐presenting cells that acquire antigens and transport them to lymphatic tissues. There are four basic ways for DC to obtain intestinal antigens: (1) presentation of antigens transported by M cells; (2) extension into the lumen through epithelial cells; (3) endocytosis of antigens by epithelial cells and (4); direct contact with antigen via functional destruction of epithelial cells.^91^ Notwithstanding that much research has been done on intestinal DCs, little is known about other intestinal antigen‐presenting cells (APCs). Interestingly, the gastrointestinal mucosa contains a large number of macrophages. Although they are thought to be coordinators of intestinal mucosal inflammation, the mechanism of their inflammatory unresponsiveness remains unclear.^92^ Recent studies have shown that intestinal macrophages, through the production of IL‐10, play an essential role in protecting the host from pathogen invasion and regulating excessive immune responses to commensal bacteria.^93^ The dysregulation of these cell functions leads to an uncontrolled intestinal immune response.^94^

### 4.2 Structure and function changes to the intestinal immune system in stroke

Intestinal immune cells play a vital role in the progression of stroke. Although the microbiota regulates immune cells in the gut, immune cells can migrate to the brain via peripheral circulation after a stroke.^73^ Intriguingly, it has been shown that mice with cerebral ischemia–reperfusion had increased differentiation of Th17 cells in the lamina propria of the small intestine (SI‐LP) after 3 days, with a concomitant increase in the expression of IL‐23 and IL‐17A in the small intestine.^95^ In contrast, the differentiation of Treg cells and the secretion of the anti‐inflammatory cytokine IL‐10 by Treg cells were decreased in the small intestine.^95^ In addition, most γδT cells in the human body mainly exist on the surface of the intestinal epithelium and participate in the innate immune response of the intestine. Interestingly, after ischemic stroke, γδT cells migrate from the gut through the peripheral circulation to the brain membrane and secrete IL‐17 into the damaged brain tissue. Subsequently, IL‐17 induces increased chemokines in the brain parenchyma, leading to massive neutrophil infiltration and exacerbating ischemic neuroinflammation.^73^ Il‐10 secreted by Treg cells in the small intestine can inhibit the differentiation of γδT cells and play a neuroprotective role.^73^ In addition to Th17/Treg or γδT /Treg responses, classical Th1 and Th2 responses may be altered after acute ischemic stroke. The researchers measured mRNA levels in the small intestine of the cytokines IFN‐γ and IL‐4, representing Th1 and Th2 effector phenotypes, respectively, and found IFN‐γ expression was increased and IL‐4 expression decreased on day 3.^95^ These findings suggested that pharmacological intervention of the immune balance of Th17/Tregs and Th1/Th2 in the small intestine could ameliorate cerebral ischemic injury.^95^

Peyer's patches represent an inductive site for immune responses mediated by T and B cells to intestinal antigens^96^, ^97^ (Figure 1). B cells are predominantly found in PPs and differentiate into IgA‐producing B cells in the presence of T cells to eliminate toxins and pathogens.^98^, ^99^, ^100^ It has been demonstrated that stress prior to cerebral ischemia can significantly reduce large intestinal IgA and bacterial translocation in a rat stroke model.^101^ In addition, dendritic cells in PPs are equally critical for initiating and developing adaptive immune responses.^99^, ^102^ It is widely thought that stroke‐induced reduction of Peyer's patches B cells and DCs could threaten local and systemic immune system homeostasis and lead to compromised local antimicrobial defenses. Interestingly, in an animal model, the number of B cells in the PPs of 129SV mice that underwent MCAO for 60 minutes was also reduced. However, the number of T cells in PPs was inconsistent with the literature. Although significant reductions were also observed, no changes were found in intestinal epithelial and lamina propria lymphocyte subsets,^103^ which may be attributed to the sensitivity of lymphocytes to stress‐induced apoptosis depending on the type and duration of stress and the phenotype and anatomical location of lymphocytes.^104^ In another study, the researchers focused on immune cell populations in the intestinal PPs one day postoperatively in mice with stroke. The results showed that the number of CD11b^+^CD11c^+^ DCs and B cells in PPs of mice after cerebral ischemia decreased. In contrast, no significant change in the number of T cells was observed.^105^

In summary, the intestinal immune system, which comprises multiple immune tissues and cells, works together in a normal physiological environment to defend against pathogenic invasion and maintain immune homeostasis. However, after a stroke, this immune homeostasis is disrupted, and the intestinal immune cells and cytokines undergo alterations. Importantly, this change is not confined to the gut but also affects distal brain tissue. The migration of lymphocytes and the increase in inflammatory mediators can exacerbate ischemic neuroinflammation, making treatment and recovery after a stroke challenging.

## 5 ENTERIC NERVOUS SYSTEM AND STROKE

### 5.1 Normal structure and function of the enteric nervous system

As the largest sensory organ in the body, the gut differs from other peripheral organs with its extensive internal nervous system called the enteric nervous system (ENS), which controls the function of the gut. The ENS originates from the embryonic neural crest and consists of the myenteric and submucosal plexuses that run nearly parallel throughout the entire gut. From an evolutionary perspective, the ENS can be considered the “first brain”.^106^ The ENS can act as a “local neural mechanism” to independently control intestinal behavior, such as regulating gastrointestinal motility, secretion, and local blood flow and interacting with the immune and endocrine systems.^107^, ^108^, ^109^ Ample evidence suggests that the interaction between intestinal epithelial cells and the ENS affects gut homeostasis. The ENS not only interacts with the microbiota, metabolites, and nutrients on the surface of intestinal epithelial cells but also with the microenvironment of immune cells and stromal cells.^110^ In addition, the ENS interacts with epithelial cells to promote barrier function and protect the intestine from pathogens in the intestine.^111^ Albeit the ENS is subject to considerable mechanical, chemical, and microbial stressors in the intestine, its structure remains stable. The ENS is stable because nearly the entire neuronal population of the ENS is continually renewed every few weeks, driven by enteric neural precursor cells (ENPCs), which continuously and rapidly generate new neurons to counteract the neuronal population lost to apoptosis.^112^ The external connection between CNS and ENS comprises sympathetic and parasympathetic nerve fibers directly connected to the gastrointestinal from the hindbrain.^113^ The central nervous system communicates with the gut through the gut‐brain axis. Vagus nerve and spinal cord sensory neurons terminate at different locations in the intestinal wall, including the muscle layer and mucosal epithelium, and play an important role in the CNS transmitting information to the ENS and vice versa.^114^

### 5.2 Structure and function changes to the enteric nervous system in stroke

Growing evidence suggests cerebral ischemia affects the enteric nervous system (Figure 1). The two main types of cerebral ischemia are focal and global. Focal cerebral ischemia refers to insufficient blood flow to specific parts of the brain, while global cerebral ischemia involves extensive brain areas. In the ENS, the neurotransmitters nitric oxide (NO) and vasoactive intestinal peptide (VIP) are thought to play essential roles in the maintenance and protection of neurons.^115^, ^116^, ^117^, ^118^ Significant enteric neuron loss has been documented in mouse ileal muscle enteric ganglia following permanent middle cerebral artery occlusion (pMCAO).^119^ The researchers further investigated the expression and the relative number of vasoactive intestinal peptide (VIP) and neuronal nitric oxide synthase (nNOS) neurons in three mouse models of pMCAO, global cerebral ischemia–reperfusion (GCIR) or chronic cerebral hypoperfusion (CCH).^120^ No changes in intestinal mucosa or muscle layer thickness were found in the three cerebral ischemia models. On day 7 after pMCAO, the ileal muscle‐enteric neurons of mice were depleted, the number of submucosal VIP‐ immunoreactive (IR) neurons increased, and the relative number of nNOS‐IR neurons did not change significantly.^120^ Intriguingly, there were no significant changes in the relative numbers of neurons or VIP and nNOS‐IR neurons in the GCIR and CCH groups, contrary to other studies where activation of the GAL‐3/TLR4 pathway led to significant neuron loss after cerebral ischemia in all three models of cerebral ischemic.^119^, ^121^, ^122^, ^123^, ^124^ However, the pMCAO model exhibited permanent damage to regional cerebral blood flow, whereas GCIR caused transient occlusion, and CCH caused a chronic decrease in central cerebral blood flow. Therefore, further damage to brain tissue may affect the neurovascular barrier and the activation of the peripheral immune system differently.

In conclusion, the transmission of information between the ENS and the central nervous system is crucial for maintaining human health. The different types of neurons in the ENS form a complex network that continuously collects and releases signals. It is well‐established that gastrointestinal dysfunction can be triggered after a stroke. In addition to the gut barrier, gut microbes, and immune system mentioned in previous articles, the enteric nervous system is also an essential factor. Loss of enteric neurons has been demonstrated in the PMCAO animal model after a stroke. However, due to the limited studies available, it can only be speculated that the loss of enteric neurons may be associated with the activation of the peripheral immune system after ischemia. The complex role of the enteric nervous system in the brain–gut axis after cerebral ischemia requires further investigation.

## 6 TREATMENT STRATEGY

The treatment of ischemic stroke remains a great challenge. Given limitations associated with the time window and surgical safety, less than 5% of stroke patients receive active and effective treatment.^125^ In recent years, immunotherapy for stroke has primarily focused on reducing injury volume and improving functional outcomes. Although the mechanism of immune‐mediated neuronal injury has attracted much attention, few drugs have reached the stage of phase II or phase III randomized controlled trials (RCT) in acute stroke. The drugs currently entering the RCT phase are recombinant interleukin‐1 receptor antagonist (IL‐1Ra) (Anakinra), anti‐ICAM‐1 antibody (Enlimomab), Minocycline, Natalizumab, and Fingolimod.^126^, ^127^, ^128^, ^129^, ^130^, ^131^ Although these drugs have shown positive results in preclinical animal stroke models, clinical trial results have not been satisfactory. Only Anakinra, Minocycline, and Fingolimod have demonstrated the potential to treat stroke in clinical studies, but further validation in large‐scale clinical trials is still needed.^132^ Preclinical studies usually use young and healthy animals without comorbidities, while in reality, most stroke patients are older, with high rates of hypertension, hyperlipidemia, and diabetes.^133^ In addition, “immunosenescence” refers to a series of age‐related changes in the immune system, which is also an important factor in treating stroke.^134^ Microglia from older brains were found to secrete more pro‐inflammatory factors and exhibited an increased tendency to polarize to the M1 phenotype compared with younger brains^135^ and a reduced ability to regenerate axons with age.^136^ Many studies have revealed that alterations in the intestinal microenvironment after ischemic brain injury induce a pro‐inflammatory immune response, enlarge the infarct size, and are strongly associated with stroke prognosis.^137^ Several treatments that modulate the gut microenvironment have shown promise in preventing or treating stroke. In the following section, we will review the impact of these treatments on stroke. We also searched for current clinical trials on treating stroke by modulating the intestinal microenvironment to provide further scientific evidence for the therapy of stroke (Table 1).

**TABLE 1:** Current clinical trials in the treatment of stroke through modulation of the intestinal microenvironment.

Interventions	Study title	Objective/methods	Trail identifier	Status
Dietary supplement: probiotics	Cognition and Gut Microbiome Associated Study of Shanghai People with Acute Ischemic Stroke	This study will collect the data from the day of admission and 3 months after stroke data and put it into analysis to provide some suggestions on the probiotics used in the clinic for the stroke patients.	NCT03812445	Recruiting
Dietary Supplement: OMNi‐BiOTiC SR‐9	A Randomized Double Blinded Placebo Controlled Study on the Effects of Dietary Supplementation with a Probiotic on Stroke Patients	Patients are recruited within seven days of stroke onset and randomly assigned to either the Control or Treatment group and subsequently take the commercially available probiotic or the placebo twice a day for 3 months.	NCT04954846	Recruiting
Dietary supplement: probiotics	A randomized controlled, prospective clinical study of probiotics in the treatment of acute ischemic stroke	The primary research purpose is to evaluate the safety and effectiveness of probiotics in treating acute ischemic stroke.	ChiCTR2100051641	Others
Buyang Huanwu decoction combined with probiotics	Study on the effect and mechanism of Buyang Huanwu decoction combined with probiotics on ischemic stroke based on intestinal flora	This study takes intestinal flora as the target and intends to clarify the effect and mechanism of Buyang Huanwu decoction combined with probiotics in the treatment of ischemic stroke to provide a new scheme for the clinical treatment of ischemic stroke.	ChiCTR2000031238	Pending
Drug: Tongfu capsules	Safety and Efficacy of the Tong‐Fu‐Xing‐Shen Herbal Formula for Stroke‐Associated Pneumonia (TFXSHF)	Stroke‐associated pneumonia (SAP) patients are recruited to clarify whether TFXS is effective and safe for the treatment of SAP and affects the immunological mechanism of the “brain–gut–lung” pathway of SAP.	NCT04275219	Recruiting
Drug: Xinglu Chengqi Decoction	Clinical research of Xinglou Chengqi Decoction on improving post‐stroke cognitive impairment due to phlegm heat and viscera excess syndrome based on brain–gut interaction	Based on brain–gut interaction, observation of the therapeutic effect of Xinglu Chengqi Decoction on post‐stroke cognitive impairment due to phlegm heat and excess viscera syndrome.	ChiCTR2000040910	Recruiting

### 6.1 Antibiotics

Although the mechanism is unclear, the gut microbiota may be a source of systemic infection in stroke patients, given that the immunosuppression initiated after stroke limits the autoimmune response.^5^, ^138^, ^139^ It is widely acknowledged that broad‐spectrum antibacterial drugs have anti‐inflammatory effects and reduce the risk of infection in stroke patients. Pre‐stroke use of amoxicillin (β‐lactam antibiotic) and clavulanic acid (β‐lactamase inhibitor) can alter immune homeostasis in the mouse small intestine by affecting the intestinal microenvironment, leading to an increase in regulatory T cells and a decrease in minus IL‐17^+^γδ T cells.^73^

Oral administration of polymyxin B modulates intestinal flora, reduces lipopolysaccharide (LPS) levels and neuroinflammation in the ischemic brain of type 2 diabetic (db/db) mice, and improves metabolic endotoxemia and stroke outcome in db/db mice.^140^ Controversially, studies on mice have shown that antibiotic‐induced changes in the intestinal flora could reduce ischemic brain damage. However, in other experiments, broad‐spectrum antibiotics caused extensive microbiota depletion and worsened stroke prognosis.^141^ Transient global forebrain ischemia (tIsc) mice given oral vancomycin or ampicillin exhibited cognitive impairment and worsened intestinal inflammation with an increase in *Enterobacter xiangfangenesis* from the Proteobacteria family.^142^ These studies indicate the intricate interconnection between commensal microbial populations and their host. Different antibiotics could induce different alterations in microbiota structure and microbiota‐derived metabolites and influence the organism's immune response. Fecal transplants were performed after pretreatment of mouse donor feces with vancomycin, streptomycin, and metronidazole. The vancomycin and streptomycin groups exhibited increased infiltration of pro‐inflammatory polymorphonuclear leukocytes and monocytes and proliferating iNKT cells in the intestinal LP.^143^ These results may be attributed to the fact that vancomycin and streptomycin treatment increased the abundance of *Bacteroides*, *Parabacteroides*, *Streptococcus*, and *unclassified Enterobacteriaceae* in the flora and led to specific enrichment of microbial metabolites such as gluconate and azelaic acid. On the other hand, the microbiota structure of the recipients receiving metronidazole‐pretreated feces showed the enrichment of *Lactobacillus* and cellobiose. These florae and metabolites could be important for modulating mucosal immunity and promoting an IL‐10‐dependent anti‐inflammatory response.^143^

### 6.2 Probiotics

Probiotics are live strains of strictly selected microorganisms that, when administered adequately, confer a health benefit on the host.^144^ Probiotics enter the gut and ensure the balance of intestinal microbes by producing antibacterial substances, competing with pathogenic microbes for epithelial adhesion and nutrients.^145^ They also provide immunomodulation by triggering a signaling cascade in epithelial cells, while their metabolites inhibit the production of bacterial toxins.^146^, ^147^ *Clostridium butyricum* pretreatment for 2 weeks has been shown to improve I/R brain injury in mice by reducing neurological deficits, alleviating oxidative stress, and inhibiting apoptosis.^148^ In another study, pretreatment with a probiotic mixture (including *Bifidobacterium breve*, *Lactobacillus casei*, *Lactobacillus bulgaricus*, and *Lactobacillus acidophilus*) for 2 weeks improved I/R mouse injury and significantly reduced brain infarct size through antioxidant mechanisms.^149^ In a mouse model of cerebral hippocampus injury, prophylactic intake of a mixture of seven probiotic bacteria reduced hippocampal neuronal damage and restored spatial memory capacity by inhibiting apoptosis.^150^ In addition, inactivated *Lactobacillus* could suppress neuronal apoptosis, reduce brain infarct volume, decrease oxidative stress, and improve neurobehavioral scores in rats by inhibiting the TLR‐4/NF‐κB signaling pathway.^151^ Although probiotics that promote intestinal microenvironmental health are mainly considered safe and valuable, abuse in specific high‐risk populations may also lead to severe infections, necessitating a risk and benefit assessment prior to use to better exploit the benefits of probiotics.^152^

### 6.3 Fecal microbiota transplantation

Fecal microbiota transplantation (FMT) enables modification of the recipient's intestinal microbiota to normalize its composition and obtain therapeutic benefits.^153^ FMT use dates back to the 4th century and was officially approved by the US Food and Drug Administration in 2013 to treat recurrent and refractory *Clostridium difficile* infections.^154^ Since then, the use of FMT has gained significant momentum, and studies in recent years have found that FMT is also efficient in treating stroke and post‐ischemic complications.^155^ After an experimental stroke, microbiota transplantation from an acute middle cerebral artery occlusion mouse model into germ‐free mice could exacerbate the lesion size and functional deficits.^5^ In contrast, mice treated with FMT from healthy donors after fMCAO exhibited significantly reduced lesion size through immunomodulatory mechanisms.^5^ Importantly, transplantation of SCFAs‐rich fecal microbiota can reshape the gut microbiota, enrich the beneficial *Lactobacillus* and repair the leaky gut, making it an effective treatment for ischemic stroke.^80^ Current evidence suggests that complications of type 2 diabetes (T2D) aggravate brain infarction in AIS. In this respect, the transplantation of feces from T2D mice supplemented with butyrate could improve the prognosis of stroke by significantly reducing infarct size and the levels of pro‐inflammatory cytokines in the serum.^156^

In addition, the “age of feces” is an essential factor to consider in FMT, as studies have found that an aging gut microbiome can reduce SCFAs in the host and lead to cognitive decline.^157^ The feces of young mice contained high levels of SCFAs‐producing bacteria and higher SCFAs concentrations, and older stroke mice that underwent young fecal transplant gavage exhibited reduced neurological deficits and inflammation, with significantly higher intestinal, brain, and plasma SCFAs concentrations.^82^ In another study, mice transplanted with an aging microbiota showed an increased ratio of Firmicutes phyla to Bacteroidetes phyla, regardless of the recipient's age. The establishment of a younger microbiota in aged mice by fecal transplantation tube feeding reduced systemic pro‐inflammatory cytokine levels and improved prognosis and survival after MCAO.^137^ Given that microbial therapy is still in its infancy, side effects such as peripheral neuropathy from unknown pathogenic organisms cannot be ruled out with the current use of FMT transplants.^158^

### 6.4 Other treatments

An increasing body of literature suggests that many herbs and their active ingredients can regulate the gut microbiota and play a key role in treating ischemic stroke at different stages.^159^, ^160^, ^161^ The efficacy of the Tanhuo decoction (THD) for AIS has been clinically proven, with THD + basic treatment in the THD group improving the efficacy of treatment for AIS by reducing LPS‐producing bacteria, significantly increasing acetic acid‐producing bacteria and enhancing the complexity of intestinal flora coexistence to inhibiting the gut microbiota‐derived metabolites LPS and TMAO compared with the basal treatment group.^162^ Xinglou Chengqi Tang (XCD) is also effective in improving neurological function in stroke mice by regulating the gut microbiota and increasing the levels of SCFAs.^163^ NaoMaiTong (NMT) can improve stroke prognosis in the MCAO rat model by protecting the intestinal barrier and modulating intestinal flora and endogenous metabolites.^164^ Tong‐Qiao‐Huo‐Xue Decoction (TQHXD) also affects the gut microbiota of stroke rats. It inhibits the excessive increase of Bacteroidetes and ameliorates the disturbance of gut microbiota after stroke. Interestingly, TQHXD also inhibited the inflammatory response induced by the peripheral immune imbalance caused by the dysregulated gut microbiota and disrupted intestinal barrier.^165^ Moreover, combined treatment of Puerariae Lobatae Radix (PLR) and Chuanxiong Rhizoma (CXR) in rats with ischemic stroke could relieve gut microbiota dysbiosis and brain–gut barrier disruption, which effectively improved neurological function, reduced cerebral infarction and alleviated complications including dyslipidemia, increased blood viscosity and thrombotic risk.^166^

In addition, bone marrow mesenchymal stem cells (BMSCs) have been shown to enhance functional recovery and improve cognitive dysfunction and neuroplasticity by regulating neurogenesis, angiogenesis, and oligodendrocyte production.^167^, ^168^ In recent years, it has been found that BMSCs can increase the abundance of *Lactobacillus* and regulate the ecological dysbiosis of the gut microbiota and promote neurological recovery as a potential treatment for ischemic stroke.^169^ Some natural products and dietary approaches also play an essential role in preventing and treating neurological diseases. They can modulate the activity of enzymes (such as kinases, regulatory receptors, and proteins) with multiple targets and signaling pathways, directly or indirectly, thus exerting neuroprotective effects.^170^, ^171^, ^172^, ^173^ These approaches may become complementary therapies in treating stroke in the future.

## 7 CONCLUDING REMARKS

In the present review, we provided a comprehensive overview of the significance of the structure and function of the intestinal environment and its interaction with ischemic stroke. While the influence of the intestinal environment on neurological function and cerebral ischemia outcomes has been established, the mechanisms underlying the regulation of brain function before and after cerebral ischemia by the intestinal environment require further investigation. Over the past decade, the combined multi‐omics analyses of genomics, transcriptomics, proteomics, and metabolomics have led to a better understanding of the gut microbiota. This review mainly collected data from studies conducted on animal stroke models and clinical studies. While most studies showed that improving the intestinal microenvironment by improving the gut microbiota is an exciting approach to treating stroke, it is still far from clinical application. In future studies, a better understanding of the impact of age and stroke comorbidity on the intestinal microenvironment will be essential to improve stroke treatment and develop new microbial therapy‐based approaches.

## AUTHOR CONTRIBUTIONS

Linna Zhao and Jie Xiao contributed equally to this work. The article is mainly conceived and written by Linna Zhao and Jie Xiao. Songlin Li performed the literature search. Yuying Guo and Rong Fu designed the figure and table. Shengyu Hua contributed to manuscript revisions. Yuzheng Du and Shixin Xu designed and supervised this work. All authors have read and approved the final submission.

## FUNDING INFORMATION

This work was supported by the National Natural Science Foundation of China (Nos. 81973626, 81774059, and 82204906), National Key Research and Development Program of China (No. 2019YFC0840709), Tianjin Municipal Science and Technology Commission of China (No. 21JCYBJC01620), and Tianjin Health Committee (No. 2021099).

## CONFLICT OF INTEREST STATEMENT

The authors declare that the research was conducted in the absence of any commercial or financial relationships that could be construed as a potential conflict of interest.

## Data Availability

Data sharing not applicable to this article as no datasets were generated or analysed during the current study.
